# Factors associated with health‐related quality of life in long‐stay inpatients with chronic schizophrenia

**DOI:** 10.1002/pcn5.42

**Published:** 2022-09-08

**Authors:** Hiroko Oyama, Kouichi Oda, Ryu Matsuo

**Affiliations:** ^1^ Department of Psychiatry Minakaze Hospital Itoshima Japan; ^2^ Department of Psychiatry Meisei Hospital Kumamoto Japan; ^3^ Department of Health Care Administration and Management, Graduate School of Medical Sciences Kyushu University Fukuoka Japan; ^4^ Center for Cohort Studies, Graduate School of Medical Sciences Kyushu University Fukuoka Japan

**Keywords:** chronic schizophrenia, extrapyramidal symptoms, inpatients, long‐stay, quality of life

## Abstract

**Aim:**

Few studies have investigated the health‐related quality of life (HRQoL) in long‐stay inpatients with chronic schizophrenia in Japan. This study aimed to clarify the factors associated with HRQoL among these patients.

**Methods:**

Out of 238 patients with chronic schizophrenia admitted to three hospitals, 101 inpatients provided informed consent to participate in the study. The patients' HRQoL was assessed using two instruments: the EuroQol 5 dimensions (EQ‐5D) as a generic index and the Subjective Well‐being Under Neuroleptic Treatment Scale, Japanese Version (SWNS‐J) as a disease‐specific index. We examined the factors associated with these HRQoL indices using multiple linear regression models.

**Results:**

The patients' mean age was 62.9 years, and 51.5% were female. The mean (standard deviation) EQ‐5D score and SWNS‐J total score were 0.776 (0.177) and 83.5 (16.5), respectively. Multiple linear regression analysis indicated that the EQ‐5D score was significantly and negatively associated with the female sex, benzodiazepine use, and Drug‐Induced Extrapyramidal Symptoms Scale scores. In contrast, the SWNS‐J total score was significantly and negatively associated with first‐generation antipsychotics use, Brief Psychiatric Rating Scale scores, Drug‐Induced Extrapyramidal Symptoms Scale scores, and Global Assessment of Functioning scale scores.

**Conclusion:**

This study identified the factors associated with two HRQoL indices among long‐stay inpatients with chronic schizophrenia in Japan. Although the analyses showed differences in the associated factors between the generic EQ‐5D and the disease‐specific SWNS‐J, extrapyramidal symptoms as adverse effects of antipsychotic treatment were found to be associated with both indices.

## Graphical abstract

Factors associated with health‐related quality of life (HRQoL) in inpatients with schizophrenia were investigated. Extrapyramidal symptoms were found to be significant factors in both the EQ‐5D as a generic index and the SWNS‐J as a disease‐specific index of subjective well‐being under antipsychotic treatment.

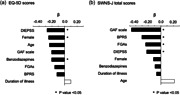

## INTRODUCTION

Schizophrenia is a complex and severely debilitating mental disorder that typically develops in late adolescence and early adulthood, and imposes heavy burdens on patients, their families, and society.^1^, ^2^ Treatment for this disorder aims not only to alleviate patients' psychiatric symptoms, but also to improve their health‐related quality of life (HRQoL). A previous study has suggested that remission in schizophrenia is associated with improved HRQoL.^3^ However, HRQoL improvement has not yet been proven to be an important predictor of symptomatic remission and functional recovery in patients with schizophrenia.^3^, ^4^, ^5^, ^6^ To understand this relationship, subjective assessments of patients (including patient‐reported outcomes) as well as objective assessments by clinicians are needed. Furthermore, such assessments are also crucial to ensuring continuous treatments that foster the clinician–patient relationship and improve medication adherence.^7^, ^8^, ^9^

Several generic and disease‐specific HRQoL instruments have been developed and successfully tested in patients with schizophrenia.^4^, ^10^, ^11^ Generic HRQoL assessments may be a valuable tool in comparisons of different patient populations, whereas disease‐specific HRQoL assessments may be more useful for evaluating specific treatment effects.^4^ It remains a matter of debate as to which instrument is more suitable for measuring HRQoL in patients with schizophrenia.^4^ In recent years, numerous studies on patients with schizophrenia have employed the EuroQol 5 dimensions (EQ‐5D) as a generic index^7^, ^12^, ^13^, ^14^, ^15^, ^16^, ^17^, ^18^ and the Subjective Well‐being Under Neuroleptic Treatment Scale (SWNS) as a disease‐specific index.^8^, ^13^, ^19^, ^20^, ^21^ Although many of these studies have reported psychotic symptoms, drug‐induced adverse effects, sex, and antipsychotic doses to be associated with HRQoL,^12^, ^15^, ^16^, ^18^, ^19^, ^20^, ^21^ others have found no such associations.^15^, ^17^, ^19^ Thus, the factors associated with HRQoL in patients with schizophrenia, especially the factors that are distinct or shared across indices, remain unclear. In addition, the majority of previous studies were conducted outside of Japan, and little is known about the factors associated with HRQoL in Japanese patients with schizophrenia.

Unlike many common physical illnesses, schizophrenia is a neuropsychiatric disorder characterized by severe impairments to social and vocational functioning, and patients often require hospital‐based care. Under Japan's previous health‐care policies and delivery systems, lengths of stay in Japanese hospitals were substantially longer than those of other Organisation for Economic Co‐operation and Development countries due to the limited availability of community‐based mental health resources.^22^ However, recent policy reformations have shifted the focus of mental health care from hospital‐based medical treatments to community‐based care, thereby resulting in a reduction of the median length of hospital stay for newly admitted patients with psychiatric disorders.^22^ Few studies have explored the factors associated with HRQoL in long‐stay inpatients with chronic schizophrenia in Japan, but a greater understanding of these factors could provide useful information to guide the promotion and improvement of community‐based care for these patients.^19^, ^23^ Thus, this study was conducted to clarify the factors associated with HRQoL in long‐stay inpatients with chronic schizophrenia in Japan.

## METHODS

### Study design and patients

In this cross‐sectional study, we examined the statistical associations between various candidate factors and HRQoL among long‐stay patients hospitalized for chronic schizophrenia. We focused on inpatients because patients with schizophrenia often require hospital‐based care. HRQoL was analyzed because it is a potentially important predictor of remission, and is also an outcome indicator of a patient's general well‐being. The initial study population comprised 258 inpatients with schizophrenia (identified using *International Statistical Classification of Diseases and Related Health Problems*, 10th Revision^24^ codes) admitted to three psychiatric hospitals in Northern Kyushu, Japan, during the study period from March to June 2020.

**Figure 1:**
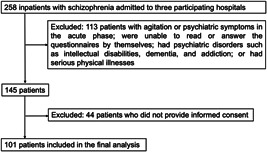
Flowchart of patient selection

Figure 1 presents a flowchart of the patient‐selection process. We excluded 113 patients who fulfilled any of the following exclusion criteria: agitation or psychiatric symptoms in the acute phase; unable to read or answer the questionnaires by themselves; psychiatric disorders, such as intellectual developmental disorders, dementia, and addiction; and serious physical illnesses. Next, we excluded 44 patients who did not provide informed consent to participate in the study. The final sample for analysis comprised 101 study patients. All study patients gave verbal and written consent to participate in the study. Responses to the questionnaires did not include patients' names to ensure anonymity. The study was approved by the ethical review committees of all three participating hospitals.

### HRQoL assessments

The 101 study patients answered questionnaires to assess HRQoL. First, HRQoL was measured using the EQ‐5D as a generic index.^25^, ^26^ Its descriptive system encompasses five dimensions: mobility, self‐care, usual activities, pain/discomfort, and anxiety/depression. Using the three‐level EQ‐5D (EQ‐5D‐3L) questionnaire, patients gave one of three response levels (no problems, some problems, and extreme problems) to each dimension. Based on these responses, each patient's EQ‐5D‐3L score was then transformed into a summary value ranging from 0.0 to 1.0.^27^ The use of the EQ‐5D was approved by the EuroQoL Research Foundation (ID34823). Next, HRQoL was measured using the Japanese version of the SWNS (SWNS‐J) as a disease‐specific index.^19^, ^28^ The SWNS‐J also encompasses five dimensions: mental functioning, self‐control, emotional regulation, physical functioning, and social integration. Using the SWNS‐J questionnaire, patients assessed their own well‐being, and a subscore was given to each dimension. The subscores were then totaled to provide a total score ranging from 0 to 120.

### Data collection

From the patients' medical records, we collected data on the following demographic and clinical characteristics: age, sex, body mass index, age of schizophrenia onset, length of hospital stay (≤1 year, 2–4 years, 5–9 years, 10–14 years, 15–19 years, and ≥20 years), educational level (senior high school or higher), presence of a key person in the family, antipsychotic dose, as well as the use of first‐generation antipsychotics (FGAs), second‐generation antipsychotics (SGAs), anticholinergics, mood stabilizers, and benzodiazepines. Antipsychotic doses were calculated as chlorpromazine (CPZ)‐equivalent doses per day based on equivalency tables for antipsychotics^29^ and long‐acting injectable antipsychotics.^30^ FGAs were defined as conventional antipsychotics, and SGAs were defined as newer antipsychotics that included risperidone and all subsequently developed antipsychotics.

In addition, we assessed psychiatric status using three psychiatric rating scales: the Brief Psychiatric Rating Scale (BPRS), the Drug‐Induced Extrapyramidal Symptoms Scale (DIEPSS), and the Global Assessment of Functioning (GAF) Scale. The BPRS, DIEPSS and GAF Scale were used to assess patients' psychiatric symptoms,^31^, ^32^ extrapyramidal symptoms as adverse effects,^33^ and general condition,^34^ respectively.

### Statistical analysis

EQ‐5D scores were categorized into quartiles as follows: Q1 (0.00–0.68), Q2 (0.69–0.75), Q3 (0.76–0.85), and Q4 (0.86–1.00). Spearman's rank correlation coefficients were calculated to analyze the correlations between the EQ‐5D, SWNS‐J, BPRS, DIEPSS, and GAF Scale.

To identify the factors associated with HRQoL, multiple linear regression models were constructed using EQ‐5D scores and SWNS‐J scores as the response variables. In all models, the following explanatory variables were included: age, sex, duration of illness (years), medication use (FGAs and benzodiazepines), and psychiatric rating scales (BPRS, DIEPSS, and GAF Scale). The variance inflation factor was used to evaluate multicollinearity. The factors associated with each dimension in the two HRQoL indices were also assessed using multiple linear regression analyses. All statistical analyses were performed using JMP Pro 15 (SAS Institute Inc.) and STATA 16 (StataCorp LP). Statistical significance was assumed for *p* < 0.05.

## RESULTS

### Patient characteristics

Table 1 summarizes the demographic and clinical characteristics of the study patients. Their mean age (standard deviation [SD]) was 62.9 (12.0) years, and 51.5% were female. The median duration of illness (interquartile range) was 36 (22–44) years, and more than half of the patients (56.4%) had been hospitalized for >5 years. Most of the patients who had been hospitalized for <5 years had long illness durations, and had been readmitted after receiving treatment for other physical diseases or transferred from other psychiatric hospitals. Among the patients, 68.3% were senior high school graduates or higher. The mean (SD) CPZ‐equivalent dose (per 100 mg/day) was 7.5 (6.2). For antipsychotics, 32.7% of the patients were using FGAs and 91.1% were using SGAs. Among the other medications, 29.7% of the patients were using anticholinergics, 45.5% were using mood stabilizers, and 65.3% were using benzodiazepines. The mean (SD) scores of the psychiatric rating scales were 47.2 (9.4) for the BPRS, 12.6 (6.1) for the DIEPSS, and 35.3 (6.4) for the GAF Scale.

**Table 1:** Demographic and clinical characteristics of the study patients

Variable	*n* = 101
Age, years, mean ± SD	62.9 ± 12.0
Female, *n* (%)	52 (51.5)
BMI, kg/m^2^, mean ± SD	22.4 ± 3.8
Duration of illness, years, median (IQR)	36 (22–44)
Educational level (senior high school or higher), *n* (%)	69 (68.3)
Key person in the family, *n* (%)	91 (90.1)
CPZ‐equivalent dose (per 100 mg/day), mean ± SD	7.5 ± 6.2
Medication use, *n* (%)	
FGAs	33 (32.7)
SGAs	92 (91.1)
Anticholinergics	30 (29.7)
Mood stabilizers	46 (45.5)
Benzodiazepines	66 (65.3)
Psychiatric rating scale	
BPRS, mean ± SD	47.2 ± 9.4
DIEPSS, mean ± SD	12.6 ± 6.1
GAF Scale, mean ± SD	35.3 ± 6.4
Length of hospital stay, *n* (%)	
≤1 year	7 (7.0)
2–4 years	37 (36.6)
5–9 years	19 (18.8)
10–14 years	11 (10.9)
15–19 years	5 (5.0)
≥20 years	22 (21.7)
Hospital, *n* (%)	
A	16 (15.8)
B	53 (52.5)
C	32 (31.7)

Abbreviations: BMI, body mass index; BPRS, Brief Psychiatric Rating Scale; CPZ, chlorpromazine; DIEPSS, Drug‐Induced Extrapyramidal Symptoms Scale; FGA, first‐generation antipsychotic; GAF, Global Assessment of Functioning; IQR, interquartile range; SD, standard deviation; SGA, second‐generation antipsychotic.

### HRQoL assessments

The distributions of the HRQoL index scores are shown (Supporting Information: Supplemental Figure I). The mean (SD) and median (interquartile range) EQ‐5D scores were 0.776 (0.177) and 0.768 (0.693–1.000), respectively. The percentages of patients who responded “no problems” to each of the five EQ‐5D dimensions were 76.2% for mobility, 83.2% for self‐care, 86.1% for usual activities, 46.5% for pain/discomfort, and 56.4% for anxiety/depression (Supporting Information: Supplemental Figure II). The mean (SD) and median (interquartile range) SWNS‐J total scores were 83.5 (16.5) and 82 (71–96) (Supporting Information: Supplemental Figure I). The mean (SD) subscores for each of the five SWNS‐J dimensions were 15.5 (4.2) for mental functioning, 18.3 (3.9) for self‐control, 16.6 (4.0) for emotional regulation, 16.0 (4.7) for physical functioning, and 17.1 (4.7) for social integration (Supporting Information: Supplemental Table I).

The correlations between the HRQoL indices and psychiatric rating scales are presented in Table 2. The correlation coefficient between the EQ‐5D and the SWNS‐J was 0.365, indicating a weak correlation. Neither the EQ‐5D nor the SWNS‐J were correlated with any of the psychiatric rating scales.

**Table 2:** Correlations between the HRQoL indices and psychiatric rating scales

	EQ‐5D	SWNS‐J	BPRS	DIEPSS	GAF Scale
EQ‐5D	1.000				
SWNS‐J	0.365	1.000			
BPRS	−0.170	−0.223	1.000		
DIEPSS	−0.202	−0.075	0.387	1.000	
GAF Scale	0.072	−0.175	−0.497	−0.569	1.000

Abbreviations: BPRS, Brief Psychiatric Rating Scale; DIEPSS, Drug‐Induced Extrapyramidal Symptoms Scale; EQ‐5D, EuroQol 5 dimensions; GAF, Global Assessment of Functioning; HRQoL, health‐related quality of life; SWNS‐J, Subjective Well‐being Under Neuroleptic Treatment Scale, Japanese version.

### Factors associated with HRQoL

**Figure 2:**
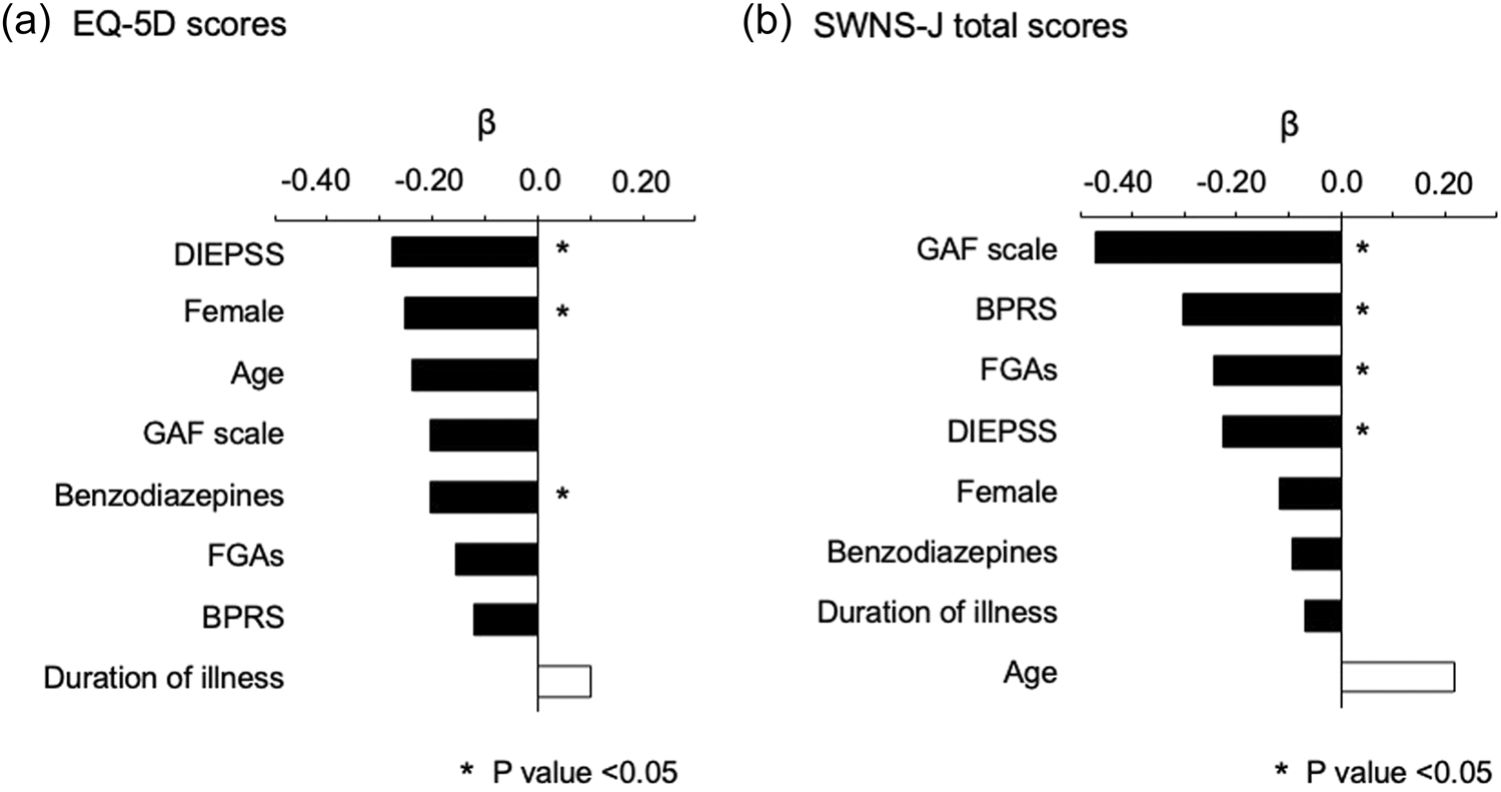
Factors associated with the health‐related quality of life (HRQoL) indices. Multiple linear regression models were constructed using (a) EQ‐5D scores and (b) Subjective Well‐being Under Neuroleptic Treatment Scale, Japanese version (SWNS‐J) scores. In all models, the following explanatory variables were included: age, sex, duration of illness (years), medication use (first‐generation antipsychotic [FGAs] and benzodiazepines), and psychiatric rating scales (Brief Psychiatric Rating Scale [BPRS], Drug‐Induced Extrapyramidal Symptoms Scale [DIEPSS], and Global Assessment of Functioning [GAF] Scale). *β* indicates standardized partial regression coefficients.

The results of the multiple linear regression analyses of the HRQoL indices are shown in Figure 2 and Table 3. The EQ‐5D score was significantly and negatively associated with the female sex, benzodiazepine use, and DIEPSS scores. The SWNS‐J total score was significantly and negatively associated with FGA use, BPRS scores, DIEPSS scores, and GAF Scale scores. The variance inflation factors indicated no multicollinearity.

**Table 3:** Multiple linear regression analyses of the HRQoL indices

	EQ‐5D		SWNS‐J		
	*β*	*P*	*β*	*P*	VIF
Age	−0.239	0.056	0.218	0.057	1.71
Female	−0.254	0.012	−0.117	0.200	1.10
Duration of illness	0.101	0.397	−0.068	0.534	1.60
FGAs	−0.154	0.111	−0.245	0.006	1.03
Benzodiazepines	−0.203	0.048	−0.092	0.324	1.15
Psychiatric rating scale					
BPRS	−0.122	0.302	−0.304	0.006	1.56
DIEPSS	−0.277	0.023	−0.227	0.041	1.61
GAF Scale	−0.204	0.125	−0.470	<0.001	1.95
Adjusted *R* ^2^	0.111		0.254		

*Note*: *β* indicates standardized partial regression coefficients.

Abbreviations: BPRS, Brief Psychiatric Rating Scale; DIEPSS, Drug‐Induced Extrapyramidal Symptoms Scale; EQ‐5D, EuroQol 5 dimensions; FGA, first‐generation antipsychotic; GAF, Global Assessment of Functioning; HRQoL, health‐related quality of life; SWNS‐J, Subjective Well‐being Under Neuroleptic Treatment Scale, Japanese version; VIF, variance inflation factor.

Table 4 presents the factors associated with each EQ‐5D dimension. Extreme problems in mobility were positively associated with age and BPRS scores. None of the explanatory variables were associated with extreme problems in the dimensions of self‐care and usual activities. In the pain/discomfort dimension, extreme problems were positively associated with the female sex. In the anxiety/depression dimension, extreme problems were positively associated with the female sex, BPRS scores, and DIEPSS scores.

**Table 4:** Multiple linear regression analyses of each EQ‐5D dimension

	Mobility	Self‐care	Usual activities	Pain/discomfort	Anxiety/depression
	*β*	*β*	*β*	*β*	*β*
Age	0.411*	0.100	0.096	0.225	0.064
Female	0.055	0.036	−0.004	0.306*	0.218*
Duration of illness	−0.062	−0.036	−0.043	−0.035	0.044
FGAs	0.187	0.121	0.164	−0.062	0.069
Benzodiazepines	0.048	−0.061	0.042	0.159	0.152
Psychiatric rating scale					
BPRS	0.290*	0.205	0.127	0.095	0.249*
DIEPSS	0.145	0.026	0.107	0.136	0.307*
GAF Scale	0.130	−0.085	0.094	0.151	0.256
Adjusted *R* ^2^	0.135	0.014	−0.034	0.067	0.125

*Note*: β indicates standardized partial regression coefficients.

Abbreviations: BPRS, Brief Psychiatric Rating Scale; DIEPSS, Drug‐Induced Extrapyramidal Symptoms Scale; EQ‐5D, EuroQol 5 dimensions; FGA, first‐generation antipsychotic; GAF, Global Assessment of Functioning.

* *p* < 0.05.

Table 5 presents the factors associated with each SWNS‐J dimension. Higher subscores in mental functioning were negatively associated with FGAs use and the scores of all three psychiatric rating scales. Higher subscores in self‐control were positively associated with age and negatively associated with FGA use. Higher subscores in emotional regulation were negatively associated with FGA use and GAF Scale scores. Higher subscores in physical functioning were negatively associated with BPRS and GAF Scale scores. Social integration scores were positively associated with age and negatively associated with GAF Scale scores.

**Table 5:** Multiple linear regression analyses of each SWNS‐J dimension

	Mental functioning	Self‐control	Emotional regulation	Physical functioning	Social integration
	*β*	*β*	*β*	*β*	*β*
Age	−0.042	0.369*	0.133	0.092	0.283*
Female	−0.073	−0.053	−0.053	−0.085	−0.168
Duration of illness	0.112	−0.008	−0.157	0.017	−0.214
FGAs	−0.257*	−0.231*	−0.212*	−0.083	−0.168
Benzodiazepines	−0.129	−0.083	−0.098	−0.006	−0.047
Psychiatric rating scale					
BPRS	−0.359*	−0.056	−0.142	−0.366*	−0.210
DIEPSS	−0.298*	−0.070	−0.198	−0.192	−0.110
GAF Scale	−0.388*	−0.235	−0.415*	−0.440*	−0.305*
Adjusted *R* ^2^	0.219	0.225	0.110	0.132	0.132

*Note*: β indicates standardized partial regression coefficients.

Abbreviations: BPRS, Brief Psychiatric Rating Scale; DIEPSS, Drug‐Induced Extrapyramidal Symptoms Scale; FGA, first‐generation antipsychotic; GAF, Global Assessment of Functioning; SWNS‐J, Subjective Well‐being Under Neuroleptic Treatment Scale, Japanese version.

* *p* < 0.05.

Next, we evaluated the impact of the different hospitals on the factors associated with the HRQoL indices. Supporting Information: Supplemental Table II presents the patient characteristics and HRQoL according to hospital. There were no differences in HRQoL among hospitals.

## DISCUSSION

This study measured HRQoL among long‐stay inpatients with chronic schizophrenia using the EQ‐5D as a generic index and the SWNS‐J as a disease‐specific index of subjective well‐being under antipsychotic treatment, and examined the factors associated with these indices. Although the HRQoL‐associated factors generally differed between the EQ‐5D and the SWNS‐J, extrapyramidal symptoms were found to be a significant factor in both indices.

### Measurement of HRQoL in long‐stay patients with chronic schizophrenia

Previous studies have reported widely varying EQ‐5D scores in patients with schizophrenia.^7^, ^15^, ^18^ For example, the mean (SD) baseline EQ‐5D scores were reported to be 0.57 (0.32) for 9340 European outpatients,^7^ 0.8 (0.2) for 60 home‐care patients in Taiwan,^15^ and 0.86 (0.13) for 153 patients in social welfare institutions in Serbia.^18^ Moreover, a Japanese study reported that mean EQ‐5D scores differed among patients with schizophrenia according to their residential environment (0.845 for group‐home residents, 0.739 for home residents, 0.723 for inpatients in an open ward, and 0.700 for inpatients in a closed ward).^14^ In our study, the mean (SD) EQ‐5D score was 0.776 (0.177), and was likely to be higher than that of inpatients admitted to general psychiatric hospitals. Participants in our study generally had longer hospitalizations and illness durations than those in previous studies.^7^, ^15^, ^18^ Because of their long‐term hospital stays, these patients may have become accustomed to their living conditions and generally satisfied with their circumstances. Elucidating the factors associated with the improvement of HRQoL in long‐stay patients with chronic schizophrenia may support the shift from hospital‐based care to home‐based care.

Relatively large proportions of inpatients in our study reported having some problems or extreme problems in the EQ‐5D dimensions of pain/discomfort and anxiety/depression, suggesting that their HRQoL may be detrimentally affected not only by physical function but also by mental function. A previous study comparing inpatients and outpatients with schizophrenia also reported that a significantly higher percentage of inpatients had problems in anxiety/depression.^14^ To support the social reintegration of patients with schizophrenia, it is necessary to clarify the specific issues and take steps to resolve them in order to provide a safe and secure living environment that can meet each individual's physical and mental needs.

In our study patients, the mean (SD) SWNS‐J total score was 83.5 (16.5). This was similar to the scores reported by previous studies, such as 72.37 (15.2) for 134 outpatients in South Korea,^21^ 73.3 (16.3) for 36 outpatients in Japan,^20^ as well as 76.5 (15.1) for 89 inpatients and 82.3 (21.3) for 39 outpatients in Japan.^19^

### Factors associated with HRQoL in long‐stay inpatients with chronic schizophrenia

In the existing literature, EQ‐5D scores have been reported to be associated with the female sex,^18^ illness durations exceeding 20 years,^7^ and higher educational levels.^18^ Although our study found the EQ‐5D to be associated with the female sex, there were no significant associations with illness duration or educational level. The adverse effects of antipsychotics (e.g., tardive dyskinesia, akathisia, pseudoparkinsonism, sexual dysfunction, sedation, and weight gain) have also been reported to be associated with lower EQ‐5D scores.^12^, ^16^ Although we did not examine these adverse effects individually, we analyzed their overall effects using the DIEPSS. Our analysis detected a significant negative association between the DIEPSS and both HRQoL indices. In a previous study on inpatients aged 65 years or older without any serious psychiatric illness, benzodiazepine use for more than 4 weeks was found to be associated with decreased HRQoL.^35^ In the EQ‐5D dimension‐level analysis, the female sex was associated with extreme problems in pain/discomfort and anxiety/depression, which suggests an association between the female sex and HRQoL.

Previous studies have reported that SWNS total scores decrease when DIEPSS scores increase.^19^, ^36^ Although our analysis found no correlation between the SWNS‐J and DIEPSS, it showed a significant and negative association between them using multiple linear regression analysis. The SWNS is indicative of cognitive and emotional functions as symptoms of schizophrenia and adverse effects of antipsychotics. The evidence that antipsychotic monotherapy switching from FGAs to SGAs (which have the same antipsychotic effects as FGAs but fewer extrapyramidal symptoms) can improve HRQoL may support our observed negative association between HRQoL and extrapyramidal symptoms.^13^, ^37^ Next, a previous study reported a positive correlation between the GAF Scale (which assesses social functioning) and the SWNS,^21^ suggesting that a higher level of social functioning is associated with higher HRQoL. In contrast, our present study showed that higher levels of social functioning were associated with lower HRQoL. The reasons for the disparity between the findings of previous studies^21^, ^38^ and those of our study are unclear. The patient populations of previous studies^21^, ^38^ have included outpatients, younger patients, and patients with shorter illness durations. Furthermore, outpatients generally have higher social functioning than inpatients. In addition, longer illness durations may be associated with lower GAF Scale scores. Thus, long‐stay inpatients may have had lower GAF Scale scores from the start of hospitalization and are unaware of their reduced social functioning, thereby giving rise to a gap between their self‐assessed capabilities and their actual capabilities. In our analysis of the factors associated with each SWNS‐J dimension, we found significant associations between higher scores in the mental functioning dimension and lower scores in the psychiatric rating scales. Accordingly, the total score of the SWNS‐J, as a disease‐specific index, may be more indicative of mental functioning. Neither age nor illness duration were associated with both HRQoL indices. Our results indicated that the psychiatric rating scales, female sex, and medication were more strongly associated with HRQoL than age or illness duration.

### Differences between HRQoL indices in patients with schizophrenia

There are various instruments available to assess HRQoL in patients with schizophrenia.^4^, ^11^ A generic index, such as the EQ‐5D, provides an overall assessment of health status and life quality but lacks information on the levels of social functioning and disease‐specific symptoms. On the other hand, a disease‐specific index, such as SWNS‐J, assesses patients' self‐perceived well‐being under antipsychotic treatment but lacks a comprehensive assessment that includes overall health status and life quality. This may explain the differences in associated factors between these two HRQoL indices, with only extrapyramidal symptoms significantly associated with both. These characteristics should be considered when such instruments are used to assess and monitor HRQoL in inpatients with schizophrenia.^4^, ^39^, ^40^

### Clinical and policy implications

Based on our findings, it may be beneficial for psychiatrists to focus on the pharmacological treatment of extrapyramidal symptoms and regularly monitor for adverse effects using the DIEPSS in stable, long‐stay inpatients with chronic schizophrenia. Furthermore, policies that introduce incentives to encourage regular DIEPSS evaluations in these patients may support the remission of schizophrenia through improvements in HRQoL. These efforts could facilitate the transition of patients from hospital‐based care to community‐based care.

Our study has several limitations. First, this was a cross‐sectional study, and causal relationships could not be ascertained. Second, the study population was relatively small as we could not obtain consent from almost one‐third of the eligible patients. Therefore, our analyses may be susceptible to biases. Third, there was limited information on the patients, and there may be unmeasured factors that affect HRQoL. Fourth, the effects of medications were not sufficiently considered in our analysis. In Japan, antipsychotic polypharmacy and the high‐dose utilization of antipsychotics have been long‐standing problems, but their reduction has progressed in recent years. In our patients, the mean number of antipsychotic drugs was 1.7 and the mean CPZ‐equivalent dose per day was 751.9 mg. These values were slightly lower than the mean number of antipsychotic drugs of 2.0 and the mean CPZ‐equivalent dose per day of 1012.3 mg, which were the initial drug‐administration regimens of patients in the Japanese government's “Safe Correction of Multidrug and High‐Dose Prescription of Antipsychotic Drugs” program.^17^ Also, we did not consider the effects of non‐pharmacological treatments. Fifth, the study used the EQ‐5D‐3L questionnaire for EQ‐5D, but this may not adequately support quantitative evaluations because its ceiling effect is reportedly higher than that of the EQ‐5D‐5L questionnaire.^41^, ^42^ Sixth, although there are a variety of instruments for measuring HRQoL and psychiatric rating scales in patients with schizophrenia, our study only focused on the EQ‐5D and the SWNS‐J. For example, the Japanese version of the Schizophrenia Quality of Life Scale is also a widely used measure to evaluate HRQoL in patients with schizophrenia.^43^ Similarly, the inclusion of an objective HRQoL score, such as the Quality of Life Scale, may help us to explore any gaps between subjective and objective scores.^44^ In addition, the Social Behavior Schedule may be a better measure than the GAF Scale for evaluating social function in inpatients.^45^ Seventh, the psychiatric rating scales were assessed by the researchers or the psychiatrist/nurse in charge, and these assessments were not blinded. Finally, it is difficult to generalize our findings to all patients with schizophrenia throughout Japan or in other countries because the study was conducted on relatively stable chronic patients admitted to three hospitals in Northern Kyushu, Japan.

In conclusion, the presence of extrapyramidal symptoms as adverse effects was associated with lower HRQoL in both the generic EQ‐5D and the disease‐specific SWNS‐J. Further studies are needed to comprehensively elucidate the factors associated with various HRQoL indices in long‐stay patients with chronic schizophrenia.

## AUTHOR CONTRIBUTIONS

Hiroko Oyama and Ryu Matsuo designed the study; conducted the analyses, interpretation of data, and drafting of the manuscript and figures. Hiroko Oyama and Kouichi Oda contributed to the acquisition of data. Ryu Matsuo made substantial contributions to the interpretation of data and revision of the text critically for important intellectual content. All authors approved the final manuscript.

## CONFLICT OF INTEREST

The authors declare no conflicts of interest.

## ETHICS APPROVAL STATEMENT

The study was approved by the ethical review committees of all three participating hospitals (Hakomatsu Hospital, Hokoda Hospital, and Meisei Hospital).

## PATIENT CONSENT STATEMENT

All patients provided written informed consent.

## CLINICAL TRIAL REGISTRATION

Clinical trial registration information is not available.

## Supporting information

Supporting information.

## Data Availability

Anonymized data are available from the corresponding author upon reasonable request.
