# Supplementary material for: Factors associated with health‐related quality of life in long‐stay inpatients with chronic schizophrenia
Source: PCN Rep. 2022 Sep 8;1(3):e42. doi: 10.1002/pcn5.42 (PMC11114288; doi:10.1002/pcn5.42)
Supplement: Supplementary file 1 — Supporting information. [file PCN5-1-e42-s001.docx]

**SUPPLEMENTAL MATERIAL**

Factors Associated with Health-Related Quality of Life in Long-Stay Inpatients with Chronic Schizophrenia

**Supplemental Figures**

Figure I. Distribution of health-related quality of life index scores

Figure II. Distribution of patient responses according to EQ-5D dimension subscores

**Supplemental Tables**

Supplemental Table I. Descriptive statistics of the SWNS-J subscores

Supplemental Table II. Demographic and clinical characteristics of the study patients according to hospital

**Supplemental Figure I. Distribution of health-related quality of life index scores**


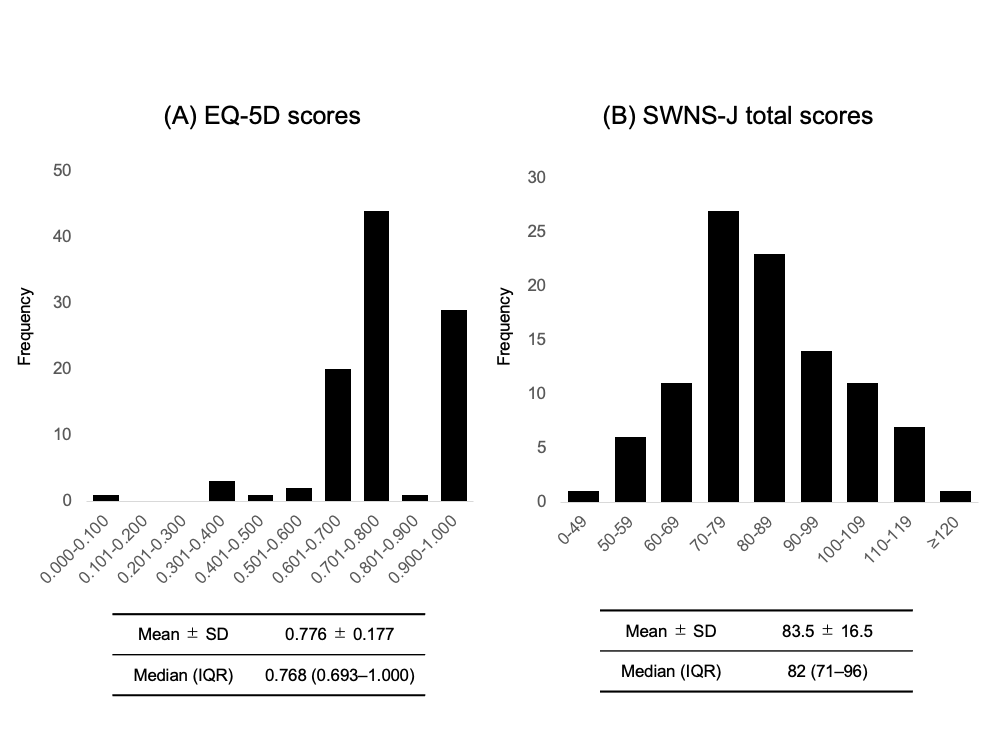


(A) EQ-5D scores and (B) SWNS-J total scores. The descriptive statistics of the scores (mean±SD and median [IQR]) are presented below each graph.

IQR, interquartile range; SD, standard deviation; SWNS-J, Subjective Well-being Under Neuroleptic Treatment Scale, Japanese version.

**Supplemental Figure II. Distribution of patient responses according to EQ-5D dimension subscores**

**
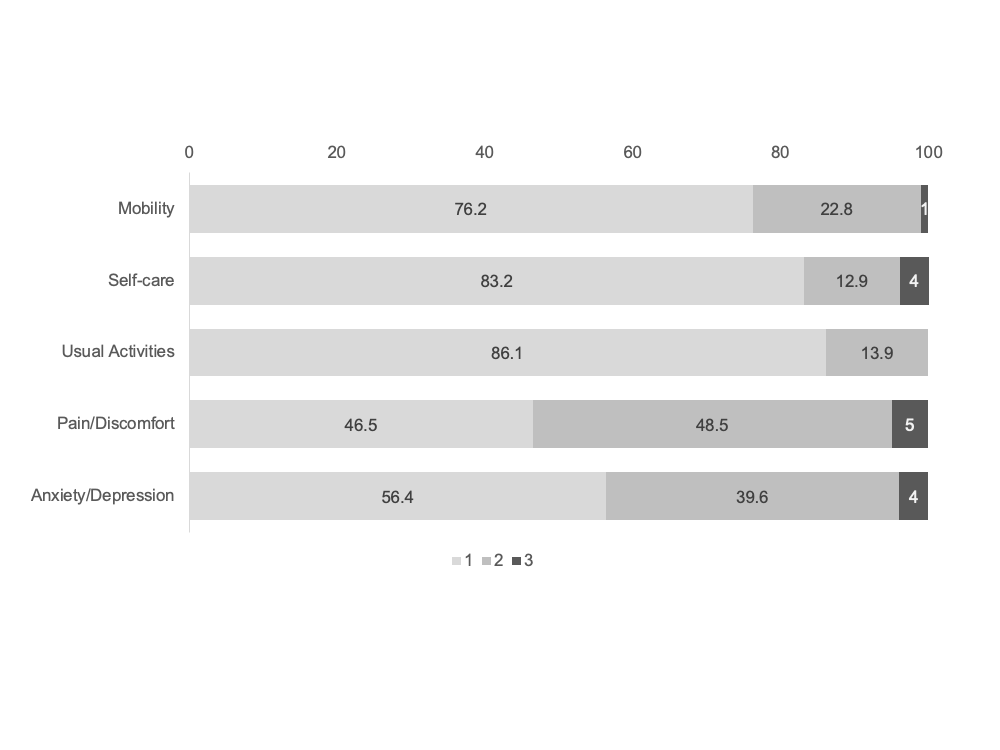
**

Level 1 represents “No problems”, Level 2 represents “Some problems”, and Level 3 represents “Extreme problems”.

Supplemental Table I. Descriptive statistics of the SWNS-J subscores

|  | N | Mean | SD | Minimum | 25% | Median | 75% | Maximum |
| --- | --- | --- | --- | --- | --- | --- | --- | --- |
| Mental functioning | 101 | 15.5 | 4.2 | 4 | 13 | 15 | 19 | 24 |
| Self-control | 101 | 18.3 | 3.9 | 9 | 15 | 18 | 21 | 24 |
| Emotional regulation | 101 | 16.6 | 4.0 | 7 | 14 | 17 | 20 | 24 |
| Physical functioning | 101 | 16.0 | 4.7 | 4 | 13 | 15 | 19 | 24 |
| Social integration | 101 | 17.1 | 4.7 | 5 | 14 | 16 | 21 | 24 |

SD, standard deviation; SWNS-J, Subjective Well-being Under Neuroleptic Treatment Scale, Japanese version.

Supplemental Table II. Demographic and clinical characteristics of the study patients according to hospital

|  | Hospital A | Hospital B | Hospital C |  |
| --- | --- | --- | --- | --- |
| Variable | n=16 | n=53 | n=32 | *P* |
| Age, years, mean±SD | 63.8±14.1 | 61.9±12.2 | 64.3±10.6 | 0.42 |
| Female, n (%) | 8 (50.0) | 29 (54.7) | 15 (46.9) | 0.78 |
| BMI, kg/m^2^, mean±SD | 21.9±3.1 | 22.0±3.3 | 23.5±4.7 | 0.36 |
| Duration of illness, years, median (IQR) | 32.4±12.0 | 31.2±15.0 | 36.0±12.1 | 0.95 |
| Educational level (senior high school or higher), n (%) | 10 (62.5) | 34 (64.2) | 25 (78.1) | 0.35 |
| Medication use, n (%) |  |  |  |  |
| FGAs | 8 (50.0) | 18 (34.0) | 7 (21.9) | 0.14 |
| SGAs | 12 (75.0) | 50 (94.3) | 30 (93.8) | 0.048 |
| Anticholinergics | 4 (25.0) | 20 (37.7) | 6 (18.8) | 0.16 |
| Mood stabilizers | 9 (56.3) | 23 (43.4) | 14 (43.8) | 0.64 |
| Benzodiazepines | 10 (62.5) | 33 (62.3) | 23 (71.9) | 0.64 |
| CPZ equivalent dose (per 100 mg/day), mean±SD | 7.7±8.4 | 8.1±6.9 | 6.5±2.9 | 0.12 |
| Psychiatric rating scale |  |  |  |  |
| BPRS, mean±SD | 42.8±11.6 | 47.7±9.7 | 48.7±7.1 | 0.06 |
| DIEPSS, mean±SD | 12.3±6.9 | 11.3±6.3 | 14.8±4.8 | 0.19 |
| GAF scale, mean±SD | 37.0±9.7 | 36.3±5.0 | 32.7±6.1 | 0.002 |
| Length of hospital stay, n (%) |  |  |  | 0.22 |
| ≤1 year | 0 (0) | 6 (11.3) | 1 (3.1) |  |
| 2–4 years | 6 (37.5) | 22 (41.5) | 9 (28.1) |  |
| 5–9 years | 5 (31.3) | 7 (13.2) | 7 (21.9) |  |
| 10–14 years | 0 (0) | 6 (11.3) | 5 (15.6) |  |
| 15–19 years | 0 (0) | 4 (7.5) | 1 (3.1) |  |
| ≥20 years | 5 (31.3) | 8 (15.1) | 9 (28.1) |  |
| HRQoL |  |  |  |  |
| EQ-5D, mean±SD | 0.718±0.195 | 0.767±0.184 | 0.819±0.148 | 0.34 |
| EQ-5D, median (IQR) | 0.768  (0.642-0.780) | 0.768  (0.705-1.000) | 0.768  (0.705-1.000) | 0.75 |
| SWNS-J, mean±SD | 85.1±17.3 | 82.2±17.3 | 84.8±14.9 | 0.64 |
| SWNS-J, median (IQR) | 82.0 (75.0-99.0) | 82.0 (70.0-94.0) | 82.5 (73.0-97.0) | 0.72 |

BMI, body mass index; BPRS, Brief Psychiatric Rating Scale; CPZ, chlorpromazine; DIEPSS, Drug-Induced Extrapyramidal Symptoms Scale; FGA, first-generation antipsychotic; GAF, Global Assessment of Functioning; HRQoL, health-related quality of life; IQR, interquartile range; SD, standard deviation; SGA, second-generation antipsychotic; SWNS-J, Subjective Well-being Under Neuroleptic Treatment Scale, Japanese version.
